# Cellulose-Chitosan-Nanohydroxyapatite Hybrid Composites by One-Pot Synthesis for Biomedical Applications

**DOI:** 10.3390/polym13101655

**Published:** 2021-05-19

**Authors:** Katia Jarquin-Yáñez, Efrain Rubio-Rosas, Gabriela Piñón-Zárate, Andrés Castell-Rodríguez, Martha Poisot

**Affiliations:** 1 Departamento de Biología Celular y Tisular, Facultad de Medicina, Universidad Nacional Autónoma de México, Circuito Interior, Ciudad Universitaria, CdMx, C.P. 04510 Coyoacan, Mexico; gabrielapinonzarate@gmail.com (G.P.-Z.); castell@unam.mx (A.C.-R.); 2 Dirección de Innovación y Transferencia de Tecnología, Benemérita Universidad Autónoma de Puebla, Prolongación de la 24 Sur y Av. San Claudio, Ciudad Universitaria, Col. San Manuel, C.P. 72570 Puebla, Mexico; efrain.rubio@correo.buap.mx; 3 Universidad del Papaloapan, Instituto de Química Aplicada, Circuito Central 200, Parque Industrial, C.P. 68301 Tuxtepec, Mexico

**Keywords:** bio-nanocomposites, nanohydroxyapatite, solvent-exchange method, human dermal fibroblast cells, bone tissue engineering

## Abstract

The development of organic–inorganic hybrid materials deserves special interest for bone tissue engineering applications, where materials must have properties that induce the survival and activation of cells derived from the mesenchyme. In this work, four bio-nanocomposites based on cellulose and variable content of chitosan, from 15 to 50 *w*% based on cellulose, with nanohydroxyapatite and β-Glycerophosphate as cross-linking agent were synthesized by simplified and low-energy-demanding solvent exchange method to determine the best ratio of chitosan to cellulose matrix. This study analyzes the metabolic activity and survival of human dermal fibroblast cells cultivated in four bio-nanocomposites based on cellulose and the variable content of chitosan. The biocompatibility was tested by the in vitro cytotoxicity assays Live/Dead and PrestoBlue. In addition, the composites were characterized by FTIR, XRD and SEM. The results have shown that the vibration bands of β-Glycerophosphate have prevailed over the other components bands, while new diffraction planes have emerged from the interaction between the cross-linking agent and the biopolymers. The bio-nanocomposite micrographs have shown no surface porosity as purposely designed. On the other hand, cell death and detachment were observed when the composites of 1 and 0.1 *w*/*v*% were used. However, the composite containing 10 *w*% chitosan, against the sum of cellulose and β-Glycerophosphate, has shown less cell death and detachment when used at 0.01 *w*/*v*%, making it suitable for more in vitro studies in bone tissue engineering, as a promising economical biomaterial.

## 1. Introduction

Bio-nanocomposites are materials containing one or more component of biological origin and some component of nanodimension; such an emerging group of materials has attracted attention on searching for optimum bioactive materials [1]. On the field of bone tissue engineering, organic–inorganic hybrid materials deserve intensive research in order to emulate the physical–chemical properties of native bone-like ideal mechanical properties and appropriate cellular responses to match the extracellular matrix of bone tissue for guiding its regeneration [2].

The biocompatibility of materials is of great importance for developing new biomedical applications; among the bio-nanocomposites developed for bone tissue engineering applications are those made from chitosan, silk fibroin and magnetite [3]. Chitosan is a cationic polysaccharide obtained by alkaline deacetylation of chitin, the principal component of crustacean exoskeleton. Due to properties such as bioactivity, biodegradability, non-toxicity and biocompatibility, chitosan has shown a wide variety of applications in tissue engineering biomaterials [4]. Additionally, chitosan has been recently gaining attention for antibacterial activity in resin-based composites for oral health [5] as well as antifungal activity [6]. Inconveniently, chitosan has shown some difficulties of swelling properties and degradation control delivering mechanical unreliability of the biomaterial [7]. On the other hand, the two main components of scaffolds that resemble bone extracellular matrix are hydroxyapatite nanoparticle (*nHAp*) and collagen nanofiber [8,9]. 

The nanocomposites of chitosan and hydroxyapatite have been shown to be superior compared to those of either *nHAp* or *Ch* alone, regarding mechanical characteristics and in vivo biomineralization [10]. Recent efforts are focused on withstanding two major limitations of bone tissue engineering materials—osteoconductivity and mechanical performance. In order to solve this issue, tri-phasic and also tetra-phasic nanocomposites have been designed by several working groups and are promising for applications.

Tetraphasic nanocomposites with uniform dispersion of *nHAp* and nano Fe_3_O_4_ by freeze-drying method into the chitosan and collagen organic matrix have been obtained. These magnetic nanocomposites were tested as bone scaffolding, demonstrating through in vitro experimentation high structural and mechanical performance for cell adhesion, proliferation, and excellent osteogenic differentiation [11].

Triphasic-nanocomposites of *Ch/nHAp* powder containing ions of Ag and Si, respectively, have been tested as antibacterial in bone substitutes, demonstrating that fine integration of Ag into the nanocomposite restricted the cytotoxic potential on cells delivering a potential bacteriostatic bone filler in the site of infected bone fracture in contrast to the higher cytotoxic effect found in the nanocomposite with Si. However, such triphasic nanocomposites synthesis is time-consuming and energy-demanding due to lyophilization in a freeze-dryer [12]. 

Another working group synthetized and characterized triphasic nanocomposites of chitosan with multiwalled carbon nanotubes to create conductive scaffolds for bone tissue engineering applications. They also followed the freeze-drying method, introducing beta-glycerophosphate as a cross-linking agent for a strong hydrogel structure formation. Even when no significant cytotoxicity was observed under the standard methyl thiazolyl tetrazolium (MTT) assay, we can say that carbon nanotubes are not yet a low-cost material for the massive production of biomedical applications [13].

In this study, we investigated the cytotoxicity and biocompatibility potential of the first time synthesized triphasic nanocomposites containing chitosan/cellulose/*nHAp* for the final purpose of guiding bone tissue regeneration. However, our approach has been to avoid energy-demanding and time-consuming synthetic methods as the works cited above; instead, the low-cost method of solvent exchange has been used here to bring compatibility between organic–inorganic components of these composites. The characterization techniques of Fourier transform infrared spectroscopy, X-ray diffraction and scanning electron microscopy were applied to obtain information about the structural and morphological features of these new materials, relevant variables of the activity and survival of cells.

The interest of using cellulose as the main part of our composites organic matrix lies on results previous reported by another working group that showed the effect of changes in nanocellulose surface chemistry groups on the proliferation and morphology of human dermal fibroblasts (hDFs). The authors reported that nanocellulose was carefully turning bioactive by reaching a threshold value on the carboxyl group density of carboxylated-Cladophora cellulose films by electrochemical TEMPO-mediated oxidation, showing cytocompatibility similar to commercial tissue culture media [14].

We expect that cellulose treated only by the solvent exchange method will bring high mechanical performance; the combination of chitosan and *nHAp* will enhance high biomineralization capacity while the beta-glycerophosphate agent will reach good dispersion of the *nHAp* in our composites.

The aim of this study was to investigate the synergy effect of cellulose/chitosan as organic matrix of theses composites on the metabolic activity and survival of hDFs.

## 2. Materials and Methods

In order to obtain composite materials by an accessible and low-cost method, we have followed the solvent exchange method applied to nanofibers of cellulose that consist of first forming a three-dimensional template through a self-assembly of the fibers, then filling the percolating architecture with a selected polymer, synthetic or natural. Before this method, it was impossible to incorporate cellulose particles into nonpolar polymers without the use of surface modification or surfactants [15,16]. Previously, we reported that the method application for the synthesis of cellulose-based hybrid nanocomposites with high content of silica nanoparticles showing inhibition growth of *Trametes versicolor* fungi [17].

The present bio-nanocomposites synthesis took the starting materials: β-Glycerophosphate disodium hydrate (*BG*), cellulose fibers of 20 µm (*C*), Chitosan powder (*Ch*) of low molecular weight and water insoluble, all provided by Sigma-Aldrich, Toluca, Mexico, and, nanohydroxyapatite (*nHAp*), own-made.

### 2.1. Nano-Hydroxyapatite, nHAp

The nanohydroxyapatite used was own-made after adjusting the well-known hydrothermal method [18] to optimal microwave conditions. The analytical grade reagents used were provided by Química-Meyer, Tlahuac, Mexico and J.T.-Baker, León, Mexico as follows: Ca(NO_3_)_2_·4H_2_O, (NH_4_)_2_HPO_4_ and CaCl_2_, respectively. The molar ratio of Ca/P was 1.67 according to the theoretical stoichiometric amount. The hydrothermal synthesis of *nHAp* was subjected to microwave radiation, in the device START D of Milestone, delivering a monophase crystalline structure of nanorod morphology, commonly found by hydrothermal methods under microwave radiation [19], with an average diameter of 47 nm measured by transmission electron microscopy [20].

### 2.2. Bio-Nanocomposites Preparation

The bio-nanocomposites were synthesized, adjusting the solvent exchange method previously reported. The first solvent exchange step was obtained adding water dropwise to *C* gently stirring to obtain a gel; then, ethanol was added dropwise at 1:1 volume ratio, still stirring during 30 min in an ultrasonic bath at 44 KHz. Then, the second solvent exchange step was applied adding acetone dropwise at 1:2 volume ratio according to water volume, stirring for up to 1 h in the ultrasonic bath, controlling the temperature up to 40 °C. Meanwhile, the *Ch* dispersion was prepared in acetone, stirring for 16 min; such dispersion was poured into the second solvent exchange step with additional stirring for 30 min more in the ultrasonic bath. The *nHAp* dispersion was prepared also in acetone stirring for 5 min; then, it was added to the previous process stirring for 45 min more in the ultrasonic bath. The *BG* dispersion in acetone (7 *w*/*v%*) was prepared under ice bath, stirring for 15 min, and at last, it was added to the process under ultrasonic bath for 1 h more. Finally, the dough-like resulting was dried at 80 °C in the oven.

The experiment was performed with four bio-nanocomposites samples reflected in Table 1, the amount of *nHAp* was kept constant while *Ch* varied from 15, 20, 25 and 50 *w*% according to *C*. The sample of highest *Ch* concentration is X1 and the opposite is X4.

### 2.3. Bio-Nanocomposites Characterization

The Fourier transform infrared (FTIR) spectra was measured in attenuated total reflectance (ATR) mode using a Bruker VERTEX 70 from 500 cm^−1^ to 4500 cm^−1^ with 40 scans per measurement in order to observe the characteristic vibrations bands of the components and also to discard the formation of new compounds. The X-ray diffraction analysis (XRD) was measured by a Bruker D8 Discover with CuKα radiation; the measurements were collected from 5 to 80 degree in 2θ in reflection mode. Scanning electron microscope (SEM) images for topography purpose were registered with secondary electrons under an acceleration voltage of 30 kV, recorded by JEOL 6610 LV coupled with elemental analyzer EDS probe by Oxford. Before measurement, the sample was sputter-coated with gold by Desk KV of Denton vacuum.

### 2.4. In Vitro Cytotoxicity

Human dermal fibroblast cells (hDFs) were isolated from skin biopsy obtained from healthy donors under informed consent. hDFs were expanded in Dulbecco’s modified Eagle´s medium with high-glucose (Life Technologies, Carlsbad, CA, USA) containing 10% fetal bovine serum (Gibco, Waltham, MA, USA), 1% antibiotic-antimycotic (100 units/mL, 100 mg/mL streptomycin, and 0.25 mg/mL amphotericin B; Life Technologies, Carlsbad, CA, USA), further mentioned as culture medium. The cells were seeded at 20,000 cells into a 75 cm^2^ flask and passaged at 80 to 90% confluence. Cell expansion and experiments were performed at 37 °C and 5% CO_2_.

For all experiments, cells were seeded at the standard density of 10,000 cells in 96-well plates and 50,000 cells in 24-well plates in culture medium with 1, 0.1 and 0.01 *w*/*v*% of each composite: X1, X2, X3 and X4.

The cell viability was assessed by the kit Live/Dead (Life Technologies, Carlsbad, CA, USA). In brief, hDFs incubated with 1, 0.1 and 0.01 *w*/*v*% of each composite for 24 h were treated with 0.12 µL of calcein and 0.5 µL of ethidium homodimer (EtHd) in 500 µL of Hanks buffer during 30 min. Calcein and EtHd detect viable and dead cells, respectively. Afterwards, the samples were analyzed on an epifluorescence microscope E80i (Olympus, Tokyo, Japan) using a 488-nm filter. All experiments were performed in triplicate at least three times.

The metabolic activity of the cells was measured after 24 h of treatment using the PrestoBlue assay (Life Technologies, Carlsbad, CA, USA). Briefly, 100 µL of measurement solution containing 10 µL of reagent and 90 µL of Hanks buffer were added to cells treated with the composites and incubated at 36 °C and 5% CO_2_ for 90 min. The 100 µL sample of the measurement solution added to cells was transferred to another 96-well plate in order to analyze their absorbance signal, measured with a spectrophotometer at an excitation wavelength of 544 nm and emission wavelength of 590 nm. For all experiments, the signal emitted by the measurement solution without cells was considered the control background. 

## 3. Results and Discussion

### 3.1. FTIR Spectrophotometry

**Figure 1:**
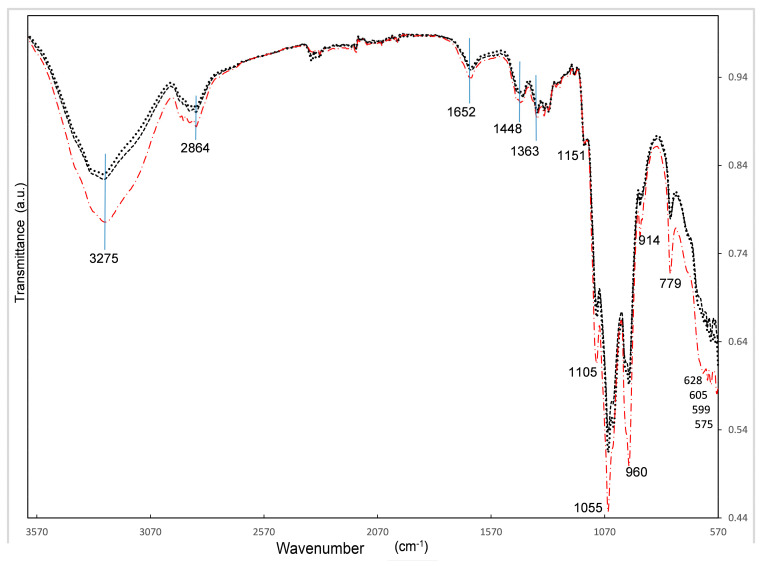
FTIR spectra of X1 (dash line), X2 (dot line) and X4 (dash dot line).

The synthesis of the biomaterials was studied by FTIR; a comparison of three such materials is found in Figure 1. X3 has been omitted due to its close similarity to X4 and for the purpose of clarity. The broad band related to the oscillation of O-H is observed between 3600 and 3000 cm^−1^. This peak shows asymmetric shape to lower wavenumbers, indicating the presence of not only hydroxyl bonds but that N-H amine groups are present in the structure also. Between 2960 and 2800 cm^−1^, several weak bands are overlapping, which could represent the stretching vibrations of aliphatic groups (-CH_n_) belonging to *C*, *Ch* and *BG*; splitting of such bands can be seen in X4, where such maxima can be related to the maximum observed in pristine *C*, *Ch* and *BG* spectra, respectively.

Around 1652 cm^−1^ there is a weak band remaining that one observed in pure *BG* in 1656 cm^−1^ that could be hiding the characteristic bands of chitosan in 1650 cm^−1^ assigned to C = O stretching of the amide bond and the one at 1590 cm^−1^ assigned to N-H bending (amide II)—neither have been observed [21]. That weak but wide band indicates that the ratio of *BG/Ch* from 2 to 6.6 is overlapping the *Ch* bands in this region. The also weak bands around 1448 cm^−1^ and 1363 cm^−1^ are assigned to deformation vibrations of -CH- and -OH, respectively [22].

Neither *nHAp* nor *BG* pristine spectra show a vibration band around 1151, but *Ch* and *C* show it. *nHAp*’s most intense band is in 1020 cm^−1^, followed by the band at 600 cm^−1^, as already observed in other works, where the 1010 to 1120 cm^−1^ bands are related to asymmetric stretching of P-O and the shoulder band at 964 cm^−1^ represents the symmetric stretching mode of P-O; also, the bands at 567 and 602 cm^−1^ are assigned to the asymmetric bending mode of O-P-O; all these vibration bands correspond to the PO_4_^3−^ group [23,24,25].

Another work that synthetized *nHAp* in silica gels has assigned the bands found at 560 to 640, 963 and 1028 to 1110 cm^−1^ to the phosphate group [26].

Several bands close together are observed at 1105, 1055, 960, 914, with the highest band in 1055 cm^−1^; again, such bands have been observed in the *BG* spectrum. The high intensity is hiding the bands of *C* and *Ch*, observed in 1026 and 1022 cm^−1^, respectively. Such vibrations are probably due to the binding of the polysaccharides characteristic CO group [27].

According to a work that prepared hydrogels of *Ch* and *BG,* the *BG* characteristic bands appeared at 1100 cm^−1^ related to aliphatic P-O-C stretching, while the band at 1050 cm^−1^ with a minor shoulder at 920 cm^−1^, the last one, may be related to –HPO_4_**^−^**; the first one is characteristic of the –PO_4_ ^2−^ group [21].

The weak peak at 779 cm^−1^ is again related to *BG* while the wide band around 600 to 630 cm^−1^ is related to *BG*, *C* and, *Ch* according to vibrations of the O = C–N group [21].

The comparison of all the bio-nanocomposite vibration band intensities allows us to relate the highest content of *BG* in X4 with the highest intensity observed. We can say that *BG* vibration bands are prevailing over *C*, *Ch* and *nHAp* characteristic bands.

### 3.2. X-ray Diffraction

**Figure 2:**
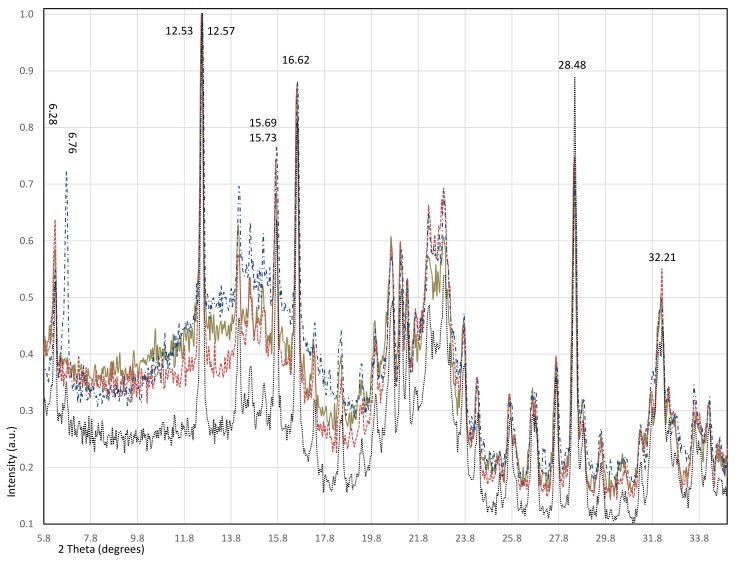
Diffractograms of X1 (gold solid), X2 (red dash), X3 (blue dash dot) and X4 (black dot).

The comparison of the four bio-nanocomposites by X-ray diffraction can be observed in Figure 2. The raw materials were identified by using the powder diffraction file database under the JCDPS card numbers: 671,540 for *Ch*, 640,738 for *HAp*, 601,502 for *C* and 050,557 for *BG* [28].

The most intense peak of *HAp*, registered in 31.77° (Miller index (121)), followed by 32.90° (Miller index (030)), are both related to the 32.2° peak of the composites since a double profile is observed. The most intense peaks of *BG* are in 16.68 and 28.40°, related to the peaks at 16.62 and 28.48° observed in the four materials. A clear depart in the position of an also high-intensity reflection is observed for X3 and X4 in 15.73°, while for X2 and X1, it is in 15.69; such a peak is also related to *BG*, which is registered in 15.61°. A similar behavior was observed in the reflection of 12.53° for X1 and X2, while for X3 and X4, it is in 12.57, this peak being the most intense in the XRD patterns. Such a peak could also be related to the *BG* peak that is found in 12.56, even when this is just a medium-high intensity reflection.

Another big contrast between the bio-nanocomposites is observed in the peak at 6.76° since it is of medium-high intensity only in X3 and of a very much low intensity in X4, while the reflection at 6.28° is observed in all the materials, related again to *BG*, which shows it at 6.26° and also around 6.7° but of very low intensity, in a similar way to X4.

Two broad regions of peaks also appear close together related to typical amorphous materials signs, e.g., *C* and *Ch*, the first with a maximum peak at 22.70° and the second at 20.73°. The first broad region registered the maximum peak at 22.87° with an additional reflection, in 22.27° for all the materials, which is new and not related to any of the raw materials. The characteristic profiles of cellulose have been observed; according to previous works, it corresponds to a mainly amorphous phase also containing Iα polymorph [29,30].

The second broad region shows the maximum peak around 21.06° being related to a small peak of *BG* in 21.08°; an additional new peak is observed in all the materials at 21.35°.

The peak around 20.6 shows differences in the maximum position for each material; in X2 and X3, it is in 20.66°; in X4, it is in 20.70°; in contrast, in X1, it lies in 20.62°. Such data is comparable with 20.73° of *Ch*. We can consider that in accordance with the highest content of chitosan in X1 the crystallographic plane is shifted to a lower angle, following the Bragg s law, giving place to a longer distance between planes, while X4 shows the angle closest to the nominal one due to the smallest content of *Ch* [31].

Another example of such a lower angle shifting is observed in the peak around 14.6° found in all samples, since it is comparable to 14.83° of *C* (Miller index (−101)); in X3 and X4, it is in 14.61°; in X1 and X2, it is in 14.58°. Peaks of similar intensity, around 14.13 and 15.1, are related to *BG*, which shows such low intensity profiles.

We can say that new planes have emerged from the interaction between the crosslinking agent, *BG*, and the biopolymers *Ch* and *C.* However, more detailed studies using, e.g., differential scanning calorimetry are needed in order to conclude it. The content of *Ch* affects such behavior since it seems that at higher content of the last one, the crystallographic planes lengthen. An exceptional behavior is displayed by the X3 material, whose content of *Ch* is only 10% of the sum of *C* and *BG*; the peak at 6.76° shows much more intensity than the peak reported in the database; unfortunately, no Miller index list is reported yet for relating such an effect with certain crystal planes modification.

### 3.3. Scanning Electron Microscopy

The comparison of micrographs of the four materials at 5000 magnification can be found in the Supplementary Materials. A close observation of the micrographs allows to see the X1 surface of high roughness, which decreases gradually with the *Ch* content up to X4 showing a rather smooth and crack-free surface.

In general, the samples have shown no porosity, as was the purpose of this work, and look very similar to each other, with the exception of X4, where particles look more homogeneous in size, around 10 µm, meaning more surface exposed of platelet-like appearance. Large, inhomogeneous elemental content is observed from the EDS results of all samples’ surfaces. In general, we can say that X1 has shown high concentration of phosphorus exposed in the surface, possibly resulting from accumulation of *BG* on there. These findings would be relevant for the final aim of this work since *BG* is not only considered an ionic cross-linker reinforcing the material final structure but also positively influences the mineralization in osteoblast cells, as reported by previous works [21,32]. 

### 3.4. In Vitro Cytotoxicity

Cell cultures are being widely used to test the toxicity of composites. The live/dead fluorescent assay provides stability, specificity, and sensitivity for assessing the proportion of live and dead cells in cell culture systems or tissues. The propidium iodide penetrate to cells that have damage in their membranes, and dead cells are detected by the fluorescence produced by the binding of propidium iodide to DNA. Calcein is metabolized in live mitochondria cells by their esterase activity, inducing a green signal [33,34].

We assessed viability of hDFs using both a live/dead assay kit and PrestoBlue staining reagent also. The PrestoBlue reagent is metabolized by living mitochondria cells, resulting in formazan blue, which can be detected at a wavelength of 570 nm by means of a spectrophotometer. This reagent directly evaluates cell viability when it comes to testing the effect of drugs or some molecules on cell cultures; indirectly, it also evaluates cell proliferation [35]. In this study, we performed both tests in order to verify the cytotoxicity results in hDFs.

**Figure 3:**
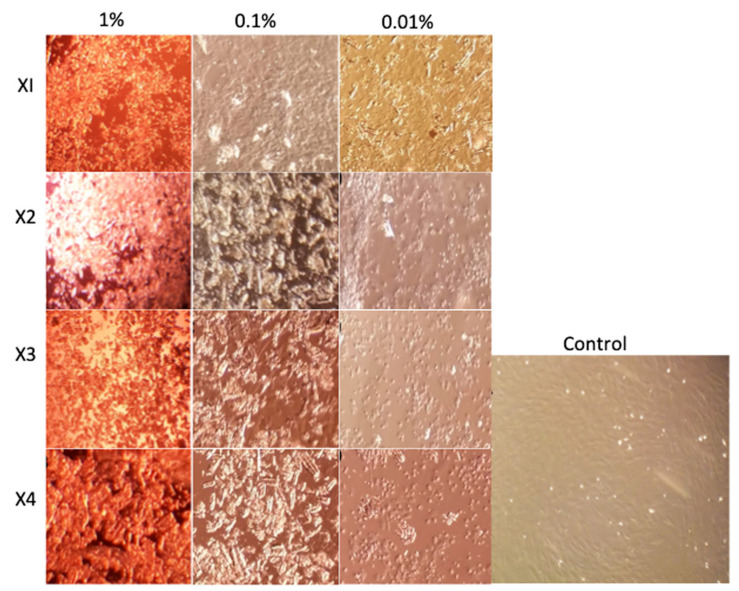
Cell cultures with concentrations of 1, 0.1 and 0.01% of the composites X1, X2, X3 and X4, in which the residues of the material were observed at higher concentrations.

After treatment of 1% composite for 24 h, fewer hDFs were found in comparison with the control group. Additionally, hDFs lost their normal morphology, characterized by a large and elongated cytoplasm, see Figure 3.

**Figure 4:**
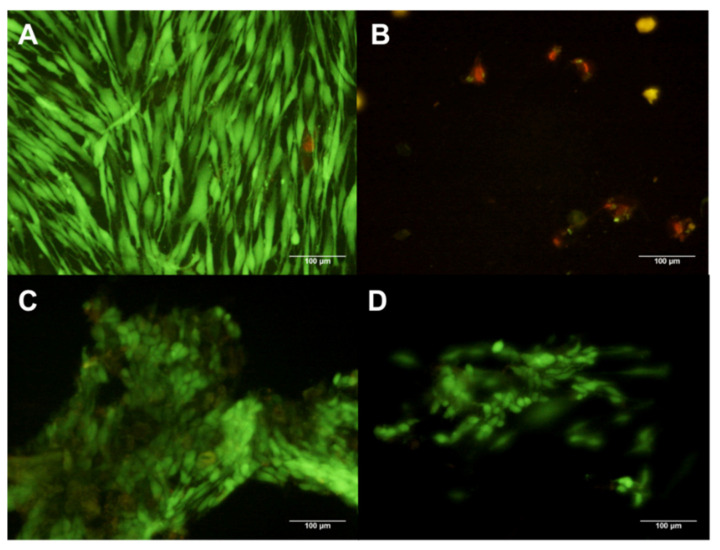
hDF cells dyed with Calcein and EtHd after 24 h of cultive with 1% (**B**) 0.1% (**C**) and 0.01% (**D**) concentration X1. The control contains no composites (**A**).

The hDF cells analyzed by Calcein (green fluorescence) and EtHd (red fluorescence), have shown that the composite X1 at 1% induced the detachment and death of hDFs (Figure 4B), while composite concentrations of 0.1% and 0.01% induced cell detachment but not cellular death (Figure 4C,D). In the control group, all hDFs were viable, having a spindle form and attached to the flask (Figure 4A).

**Figure 5:**
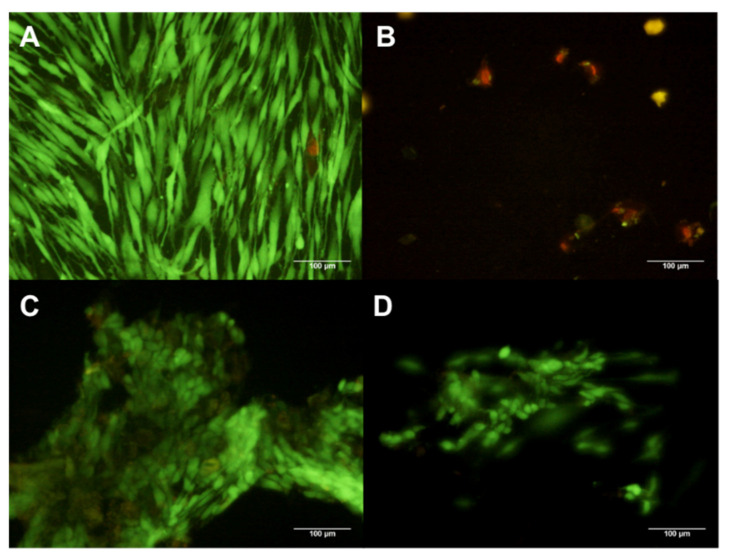
hDF cells dyed with Calcein and EtHd after 24 h of cultive with 1% (**B**) 0.1% (**C**) and 0.01% (**D**) concentration X2. The control contains no composites (**A**).

In the case of composite X2, using a 1% and 0.1% concentration on hDFs induced detachment and cell death (Figure 5B,C); in contrast, 0.01% concentration of the composite induced no changes (Figure 5D) in comparison with untreated hDFs (Figure 5A).

**Figure 6:**
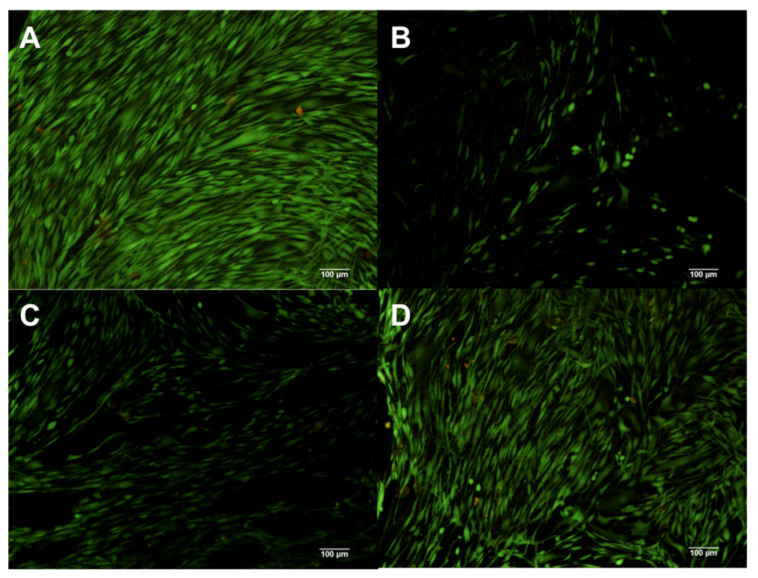
hDF cells dyed with Calcein and EtHd after 24 h of cultive with 1% (**B**) 0.1% (**C**) and 0.01% (**D**) concentration X3. The control contains no composites (**A**).

The composite X3 at 1% and 0.1% induced a diminished number of cells; however, all were viable (Figure 6B,C). In addition, close similarity was observed in hDFs cells treated with 0.01% of the composite X3 in comparison with the control group (Figure 6A,D).

**Figure 7:**
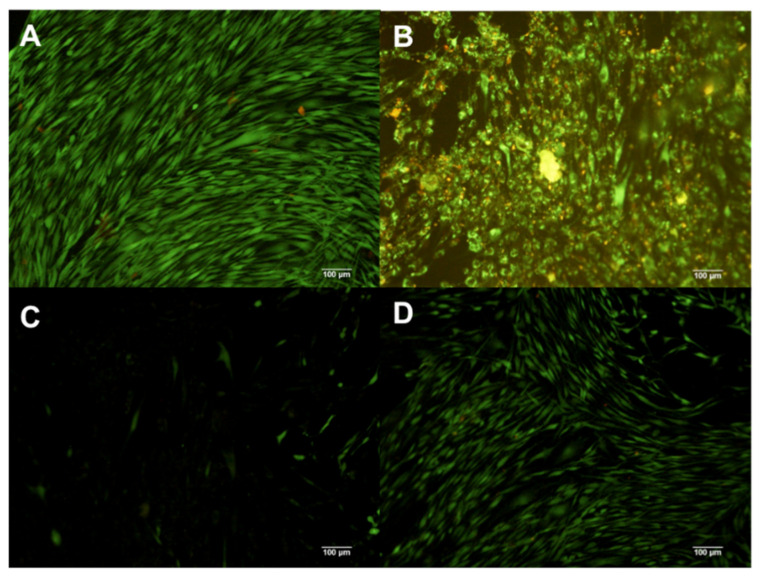
hDF cells dyed with Calcein and EtHd after 24 h of cultive with 1% (**B**) 0.1% (C) and 0.01% (**D**) concentration X4. The control contains no composites (**A**).

The composite X4 at 1% induced cell death of almost all cells; nevertheless, all cells were attached to the flaks (Figure 7B). The group treated with 0.1% concentration presented a low number of viable cells attached to the flask (Figure 7C). In contrast, hDFs exposed to the 0.01% concentration (Figure 7D) displayed no big differences compared with the control group (Figure 7A).

These results have shown that the maximum concentration at which composites X2, X3 and X4 can be used is 0.01%, since such a dose does not induce cell detachment and death. In the case of the X1 composite, it remains to be established which is the maximum concentration at which there is cell viability.

In this study, hDFs were used and not osteoblasts or mesenchymal stem cells because the objective was to establish the maximum concentration in which these composites can be used. In a later study, the osteoinductive and osteoconductive capacity of these composites in mesenchymal stem cells will be analyzed.

To evaluate the cell metabolism of hDFs, the experiments were performed with composites at 0.01% and the PrestoBlue assay was carried out also.

**Figure 8:**
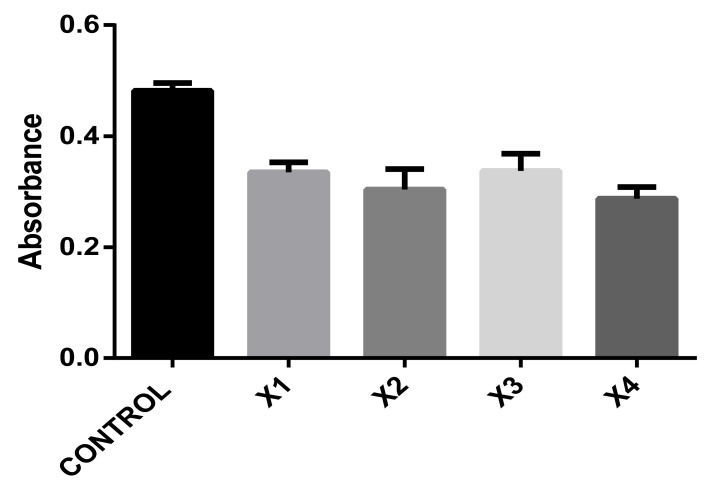
Absorbance graph of PrestoBlue reagent of hDFs seeded with 0.01% of every composite X1, X2, X3 and X4.

A significant decrease in hDFs metabolism was observed, even when no differences between groups were found, see Figure 8.

## 4. Conclusions

A low-energy-demanding one-pot method of bio-nanocomposite synthesis was developed to obtain four materials based on cellulose, chitosan, nanohydroxyapatite and β-glycerophosphate disodium hydrate.

The FTIR, XRD and SEM characterization techniques applied corroborated the fine interaction between the components for delivering four new bio-nanocomposites of variable chitosan content against the cellulose matrix. A clear split in the materials’ features was detected for X1 and X2 to differentiating from X3 and X4, which directly affected the biocompatibility performance. Some other characterization techniques can be applied for the future experiments, such as water uptake and thermal-gravimetric analysis.

The cytotoxicity was tried by in vitro experiments to determine that the composite behavior is very similar since cell death and detachment were observed at concentrations of 1% and 0.1% in all samples. It is noteworthy that all composites at concentrations of 0.01% have shown good viability and did not have significant differences between them. A significant difference was observed with respect to the control, which showed more cell survival than the composites experiments.

However, the composite X3 showed less cell death and detachment at those concentrations; it is necessary to perform more in vitro experiments considering other concentrations and also using polymeric scaffoldings as a substrate of this composite for the purpose of comparison. On the other side, composite X1, which has shown a high concentration of phosphorous on its surface, is the one that induced the most death and detachment of cells.

After confirming the physical characteristics and biocompatible doses of the bio-composites, the next step should be to carry out studies related to the effect of such composites on the differentiation of mesenchymal cells to osteoblasts.

Another further work would be to design the bio-nanocomposites addition to well-known tissue engineering scaffolds such as gelatin-hyaluronic acid to evaluate the differentiation of stem cells to bone cells.

## Figures and Tables

**Table 1:** Composition of bio-nanocomposites prepared.

Label	*C* g	*BG* g	*Ch* g	*Ch/C + BG%*	*BG/Ch*	*nHAp* g
X1	5	5	2.5	25	2	0.5
X2	5	5	1.25	12.5	4	0.5
X3	5	5	1	10	5	0.5
X4	5	5	0.75	7.5	6.66	0.5

## Data Availability

The data presented in this study are available on request from the corresponding author.

## Supplementary Materials

The following are available online at https://www.mdpi.com/article/10.3390/polym13101655/s1, Figure S1: SEM images of X1, X2, X3 and X4 under 5000 magnification.

## References

[1] Darder M., Aranda P., Ruiz-Hitzky E. (2007). Bionanocomposites: A new concept of ecological, bioinspired, and functional hybrid materials. Adv. Mater..

[2] Catauro M., Renella R.A., Papale F., Vecchio Ciprioti S. (2016). Investigation of bioactivity, biocompatibility and thermal behavior of sol–gel silica glass containing a high PEG percentage. Mater. Sci. Eng. C Mater Biol. Appl..

[3] Aliramaji S., Zamanian A., Mozafari M. (2017). Super-paramagnetic responsive Silk Fibroin/chitosan/magnetite scaffolds with tunable pore structures for bone tissue engineering applications. Mater. Sci. Eng. C Mater. Biol. Appl..

[4] Salgado-Delgado A., Hernández-Cocoletzi H., Rubio-Rosas E., Escobedo-Morales A., Chigo-Anota E., Olarte-Paredes A., Salgado-Delgado R., Trejo-Duran M., Castaño V. (2019). Electrospinning production of PVA/CS/HEMA/nHA bionanocomposite. Int. J. Nano Biomater..

[5] Ali S., Sangi L., Kumar N., Kumar B., Khurshid Z., Zafar M.S. (2020). Evaluating antibacterial and surface mechanical properties of chitosan modified dental resin composites. Technol. Health Care.

[6] Husain S., Al-Samadani K.H., Najeeb S., Zafar M.S., Khurshid Z., Zohaib S., Qasim S.B. (2017). Chitosan biomaterials for current and potential dental applications. Materials.

[7] She Z., Zhang B., Jin C., Feng Q., Xu Y. (2008). Preparation and in vitro degradation of porous three-dimensional silk fibroin/chitosan scaffold. Polym. Degrad. Stab..

[8] Tavakol S., Ragerdi Kashani I., Azami M., Khoshzaban A., Tavakol B., Kharrazi S., Ebrahimi S., Rezayat Sorkhabadi S.M. (2012). In vitro and in vivo investigations on bone regeneration potential of laminated hydroxyapatite/gelatin nanocomposite scaffold along with DBM. J. Nanopart. Res..

[9] Sionkowska A., Kozlowska J. (2010). Characterization of collagen/hydroxyapatite composite sponges as a potential bone substitute. Int. J. Biol. Macromol..

[10] Tavakol S., Khoshzaban A., Azami M., Kashani I.R., Tavakol H., Yazdanifar M., Sorkhabadi S.M. (2013). The effect of carrier type on bone regeneration of demineralized bone matrix in vivo. J. Craniofac. Surg..

[11] Zhao Y., Fan T., Chen J., Su J., Zhi X., Pan P., Zou L., Zhang Q. (2019). Magnetic bioinspired micro/nanostructured composite scaffold for bone regeneration. Colloid Surface B.

[12] Tavakol S., Nikpour M.R., Hoveizi E., Tavakol B., Rezayat S.M., Adabi M., Abokheili S., Jahanshahi M. (2014). Investigating the effects of particle size and chemical structure on cytotoxicity and bacteriostatic potential of nano hydroxyapatite/chitosan/silica and nano hydroxyapatite/chitosan/silver; as antibacterial bone substitutes. J. Nanopart. Res.

[13] Gholizadeh S., Moztarzadeh F., Haghighipour N., Ghazizadeh L., Baghbani F., Shokrgozar M.A., Allahyari Z. (2017). Preparation and characterization of novel functionalized multiwalled carbon nanotubes/chitosan/β-Glycerophosphate scaffolds for bone tissue engineering. Int. J. Biol. Macromol..

[14] Hua K., Rocha I., Zhang P., Gustafsson S., Ning Y., Strømme M., Mihranyan A., Ferraz N. (2016). Transition from Bioinert to Bioactive Material by Tailoring the Biological Cell Response to Carboxylated Nanocellulose. Biomacromolecules.

[15] Capadona J.R., Van Den Berg O., Capadona L.A., Schroeter M., Rowan S.J., Tyler D.J., Weder C. (2007). A versatile approach for the processing of polymer nanocomposites with self-assambled nanofibre templates. Nat. Nanotechnol..

[16] Eichhorn S.J., Dufresne A., Aranguren M., Marcovich N.E., Capadona J.R., Rowan S.J., Weder C., Thielemans W., Roman M., Renneckar S. (2010). Current international research into cellulose nanofibres and nanocomposites. J. Mater. Sci..

[17] Rodríguez-Robledo M.C., González-Lozano M.A., Ponce-Peña P., Quintana Owen P., Aguilar-González M.A., Nieto-Castañeda G., Bazán-Mora E., López-Martínez R., Ramírez-Galicia G., Poisot M. (2018). Cellulose-Silica Nanocomposites of High Reinforcing Content with Fungi Decay Resistance by One-Pot Synthesis. Materials.

[18] Chen F., Huang P., Zhu Y.J., Wu J., Zhang C.L., Cui D.X. (2011). The photoluminescence, drug delivery and imaging properties of multifunctional Eu3+/Gd3+ dual-doped hydroxyapatite nanorods. Biomaterials.

[19] Lak A., Mazloumi M., Mohajerani M.S., Zanganeh S., Shayegh M.R., Kajbafvala A., Arami H., Sadrnezhaad S.K. (2008). Rapid Formation of Mono-Dispersed Hydroxyapatite Nanorods with Narrow-Size Distribution via Microwave Irradiation. J. Am. Ceram. Soc..

[20] Delgado Jiménez J.F. (2013). Síntesis de Nanopartículas de Hidroxiapatita Dopada con Eu3+ por Irradiación de Microondas. Bacherol’s Thesis.

[21] Skwarczynska A., Kaminska M., Owczarz P., Bartoszek N., Walkowiak B., Modrzejewska Z. (2018). The structural (FTIR, XRD, and XPS) and biological studies of thermosensitive chitosan chloride gels with β-glycerophosphate disodium. J. Appl. Polym. Sci..

[22] Tabaght F.E., El Idrissi A., Bellaouchi R., Asehraou A., Aqil M., El Barkany S., Benarbia A., Achalhi N., Tahani A. (2020). Cellulose grafted aliphatic polyesters: Synthesis, characterization and biodegradation under controlled conditions in a laboratory test system. J. Mol. Struct..

[23] Lin L., Hao R., Xiong W., Zhong J. (2015). Quantitative analyses of the effect of silk fibroin/nano-hydroxyapatite composites on osteogenic differentiation of MG-63 human osteosarcoma cells. J. Biosci. Bioeng..

[24] Pascu E.I., Stokes J., McGuinness G.B. (2013). Electrospun composites of PHBV, silk fibroin and nano-hydroxyapatite for bone tissue engineering. Mater. Sci. Eng. C Mater Biol. Appl..

[25] Mobika J., Rajkumar M., Nithya Priya V., Linto Sibi S.P. (2020). Substantial effect of Silk fibroin reinforcement on properties of Hydroxyapatite/Silk fibroin nanocomposite for bone tissue engineering application. J. Mol. Struct..

[26] Rivera-Munoz E.M., Fazel R. (2011). Hydroxyapatite-Based Materials: Synthesis and Characterization, Biomedical Engineering—Frontiers and Challenges.

[27] Pereira R.M., Andrade G.S.S., Castro H.F.D., Campos M.G.N. (2017). Performance of chitosan/glycerol phosphate hydrogel as a support for lipase immobilization. Mat. Res..

[28] Gates-Rector S., Blanton T. (2019). The Powder Diffraction File: A Quality Materials Characterization Database. Powder Diffr..

[29] French A.D. (2014). Idealized powder diffraction patterns for cellulose polymorphs. Cellulose.

[30] Fawcett T.G., Crowder C.E., Kabekkodu S.N., Needham F., Kaduk J.A., Blanton T.N., Petkov V., Bucher E., Shpanchenko R. (2013). Reference materials for the study of polymorphism and crystallinity in cellulosics. Powder Diffr..

[31] Hammond C. (1998). The Basics of Crystallography and Diffraction, IUCr Texts on Crystallography, No 3.

[32] Niranjan R., Koushik C., Saravanan S., Moorthi A., Vairamani M., Selvamurugan N. (2013). A novel injectable temperature-sensitive zinc doped chitosan/β-glycerophosphate hydrogel for bone tissue engineering. Int. J. Biol. Macromol..

[33] Matoug-Elwerfelli M., Nazzal H., Raif E.M., Wilshaw S.P., Esteves F., Duggal M. (2020). Ex-vivo recellularisation and stem cell differentiation of a decellularised rat dental pulp matrix. Sci. Rep..

[34] Pfeffer B.A., Fliesler S.J. (2017). Streamlined duplex live-dead microplate assay for cultured cells. Exp. Eye Res..

[35] Ayyagari V.N., Diaz-Sylvester P.L., Jeff Hsieh T.H., Brard L. (2017). Evaluation of the cytotoxicity of the Bithionol-paclitaxel combination in a panel of human ovarian cancer cell lines. PLoS ONE.

