# Supplementary material for: Cellulose-Chitosan-Nanohydroxyapatite Hybrid Composites by One-Pot Synthesis for Biomedical Applications
Source: Polymers (Basel). 2021 May 19;13(10):1655. doi: 10.3390/polym13101655 (PMC8161035; doi:10.3390/polym13101655)
Supplement: Supplementary file 1 [file polymers-13-01655-s001.zip › polymers-1209651-supplementary.pdf]

polymers

MDPI

Supplementary Material: Cellulose-Chitosan-Nanohydroxyapatite Hybrid Composites by One-Pot Synthesis for Biomedical Applications

Katia Jarquin-Yáñez, Efrain Rubio-Rosas, Gabriela Piñón-Zárate, Andrés Castell-Rodríguez, Martha Poisot

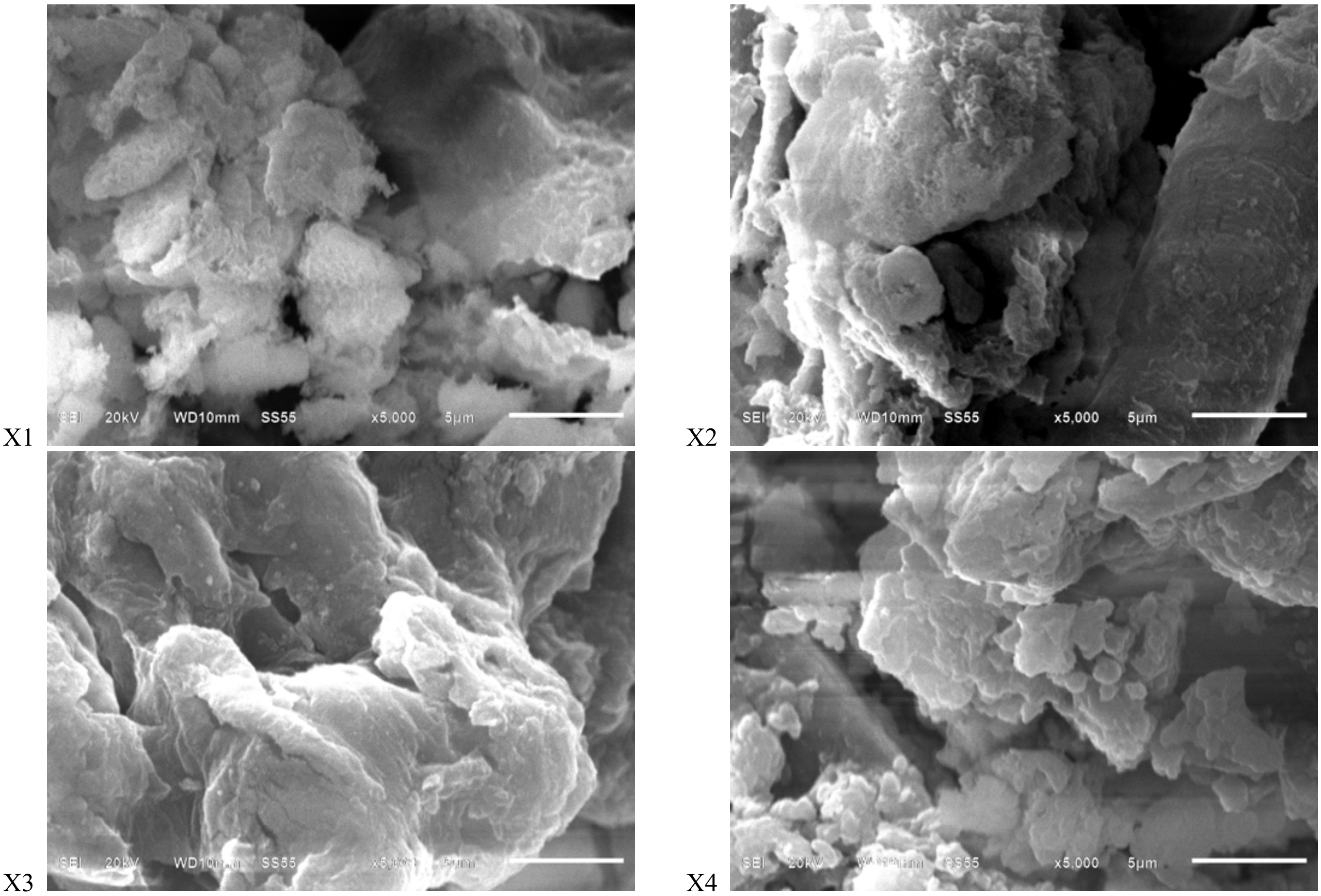

Figure S1. SEM images of X1, X2, X3 and X4 under 5000 magnification.
